# Entropy Generation-Based Assessment of Thermodynamic Irreversibility in Turbulent Conjugate Heat Transfer Systems Under Realistic Boundary Conditions

**DOI:** 10.3390/e28050573

**Published:** 2026-05-20

**Authors:** Bekir Dogan

**Affiliations:** Department of Machinery and Metal Technologies, Tokat Vocational School, Tokat Gaziosmanpaşa University, Tokat 60100, Türkiye; bekir.dogan@gop.edu.tr; Tel.: +90-544-423-82-93

**Keywords:** entropy generation, conjugate heat transfer, turbulent pipe flow, thermodynamic irreversibility, Bejan number, Biot number, energy efficiency

## Abstract

**Featured Application:**

The results of this study can support the thermodynamic design and optimization of engineering systems involving conjugate heat transfer, such as industrial piping, heat exchangers, and energy transport lines. The entropy-based analysis provides practical guidance for minimizing thermodynamic irreversibility and improving energy efficiency under realistic operating conditions, particularly by optimizing wall properties and external heat transfer characteristics.

**Abstract:**

Entropy generation analysis provides a thermodynamic framework for quantifying irreversibility in thermal systems. However, most existing second-law studies rely on simplified boundary conditions and do not consider fully coupled conjugate heat transfer involving fluid convection, wall conduction, and external heat exchange. Consequently, thermodynamic assessments under realistic conditions remain limited. This study presents an entropy generation-based assessment of turbulent conjugate heat transfer in circular pipes by considering the combined effects of wall thickness ratio (0.02–0.08), wall thermal conductivity (0.2–400 W/m·K), and external convection (5–100 W/m^2^·K). A three-dimensional steady RANS-based conjugate heat transfer model is employed, and entropy generation is evaluated to quantify irreversibility within fluid and solid domains. The results indicate that wall-related thermal resistances significantly affect thermodynamic performance. Variations in wall conductivity lead to approximately 15–20% changes in total irreversibility, while increasing external convection from 5 to 20 W/m^2^·K results in up to 25–30% variation. Increasing wall thickness enhances conductive entropy generation, whereas higher Reynolds numbers increase overall irreversibility. These findings demonstrate that the Biot number is a key parameter governing irreversibility distribution. The results provide energy-efficient design insights for optimizing thermally coupled engineering systems under realistic operating conditions.

## 1. Introduction

Thermal fluid devices such as piping networks, heat exchangers, and energy systems are traditionally designed and evaluated using first-law performance indicators, including heat transfer rate, Nusselt number, and pressure drop. While these metrics effectively quantify thermal capacity and hydraulic penalties, they do not explicitly capture the thermodynamic cost associated with irreversible transport processes. In practical systems, finite temperature gradients, viscous friction, and coupled heat-transfer pathways inevitably generate irreversibilities that degrade the quality and efficiency of energy utilization. These irreversibilities can be rigorously quantified through entropy generation analysis based on the second law of thermodynamics. Within this framework, the entropy generation minimization (EGM) method, originally introduced by Bejan, has become a fundamental tool for thermodynamic assessment and optimization of finite-size thermal systems by simultaneously accounting for heat-transfer and fluid-friction losses [1].

In internal forced convection, total entropy generation is commonly decomposed into two primary contributions: (i) entropy generation due to heat transfer across finite temperature gradients and (ii) entropy generation associated with viscous dissipation caused by fluid friction. The relative dominance of these mechanisms strongly depends on flow conditions and thermal boundary constraints. Increasing Reynolds number typically intensifies frictional irreversibility due to enhanced velocity gradients, whereas stronger convective heat transfer often increases thermal irreversibility through steeper temperature gradients. This competition between thermal and mechanical irreversibility mechanisms is frequently characterized using the Bejan number, which represents the fraction of total entropy generation attributed to heat-transfer irreversibility. Numerous studies on turbulent internal flows report a monotonic decrease in the Bejan number with increasing Reynolds number, indicating that improved convective performance may be accompanied by increased thermodynamic losses and highlighting the necessity of balanced thermo-hydraulic optimization rather than heat-transfer enhancement alone [2].

A substantial body of literature has applied entropy generation analysis to internal flow configurations such as smooth tubes, enhanced surfaces, and insert-assisted passages [3,4,5]. These investigations demonstrate that second-law-based metrics can significantly alter conclusions derived solely from first-law indicators by revealing trade-offs between heat-transfer enhancement and frictional penalties. However, the majority of these studies assume idealized thermal boundary conditions, such as prescribed wall temperature or uniform heat flux. Such assumptions neglect the fact that many real engineering systems operate under conjugate heat transfer (CHT) conditions, where internal convection interacts with heat conduction through the wall and external heat exchange with the surrounding environment.

Conjugate heat transfer effects become particularly important in circular pipe systems when wall thickness and wall material conductivity are non-negligible or when pipes exchange heat with ambient surroundings through external convection. Under these realistic conditions, the overall thermal resistance is distributed among internal convective resistance, conductive resistance within the pipe wall, and external convective resistance. Variations in any of these components significantly modify temperature gradients in both fluid and solid domains, influencing not only heat-transfer performance but also the magnitude and spatial distribution of thermodynamic irreversibilities. Previous studies have shown that wall conduction and external boundary conditions can strongly affect temperature development in thick-walled cylindrical pipes [6,7,8,9]. Nevertheless, most second-law investigations still rely on simplified wall boundary assumptions, limiting their ability to attribute entropy generation to specific physical layers and to quantify how external convection reshapes the entropy-generation budget of fully coupled systems.

Recent research has extended entropy generation analysis to selected multiphysics and conjugate configurations [10,11,12]. Despite these advances, systematic second-law assessment of turbulent conjugate heat transfer in circular pipe systems under realistic external convection remains largely unexplored in the open literature. In particular, the combined influence of wall thickness, wall material conductivity, and external convective heat-transfer intensity on (i) the partition of entropy generation between heat-transfer and frictional mechanisms and (ii) the redistribution of irreversibility between fluid and solid domains has not been comprehensively quantified over a broad parameter space representative of practical engineering applications.

Motivated by this gap, the present study presents a comprehensive second-law analysis of turbulent conjugate heat transfer in circular pipe systems while explicitly accounting for variations in (a) wall thickness ratio, (b) wall material thermal conductivity, and (c) external convective heat-transfer coefficient. The analysis is based on a fully coupled three-dimensional conjugate heat-transfer framework and a systematic parametric dataset in which Reynolds number, wall thickness ratio, wall conductivity, and external convection conditions are varied to represent realistic piping and heat-exchanger operating environments [13]. Entropy generation metrics are obtained through post-processing of validated conjugate heat-transfer solutions, enabling consistent and direct thermodynamic comparison across all investigated cases. Unlike our previous first-law-based study, the present work adopts a fundamentally different thermodynamic perspective by focusing on entropy generation and irreversibility distribution under fully coupled conjugate heat transfer conditions. It should be emphasized that no additional numerical simulations were performed; instead, the previously validated dataset is reinterpreted within a second-law framework to extract fundamentally different physical insights. Therefore, the present study constitutes an independent thermodynamic analysis rather than an extension of previously reported results.

The main contributions of the present study can be summarized as follows:

- Quantification and decomposition of total entropy generation in turbulent conjugate heat transfer within circular pipe systems over a wide operational parameter range;
- Domain-wise evaluation of irreversibility by distinguishing entropy generation in fluid and solid regions;
- Second-law regime identification using the Bejan number to complement first-law performance metrics;
- Thermodynamic interpretation of external convection as a controllable boundary resistance influencing entropy-generation distribution;
- Entropy-based design guidance for thermodynamic optimization of real engineering pipe systems.

The remainder of the paper is organized as follows. Section 2 presents the physical model and governing equations for entropy generation. Section 3 describes the numerical framework and post-processing methodology. Section 4 discusses second-law performance results and parametric effects. Finally, Section 5 summarizes the findings and outlines practical implications for thermodynamically informed design of conjugate pipe systems.

## 2. Physical Model and Governing Equations for Entropy Generation

The present analysis is based on a fully coupled conjugate heat transfer (CHT) framework, in which turbulent forced convection in the fluid region is solved simultaneously with heat conduction in the pipe wall and convective heat exchange at the external surface. Under steady-state operating conditions, entropy generation arises from irreversible transport processes associated with finite temperature gradients during heat transfer and viscous dissipation due to fluid friction. In accordance with the second law of thermodynamics, the total entropy generation rate within the system can be expressed as the sum of thermal and mechanical contributions [1,4].

### 2.1. Total Entropy Generation Rate

For a control volume encompassing both the fluid domain and the solid pipe wall, the total entropy generation rate is defined as

(1) $${\dot{S}}_{gen,total}={\dot{S}}_{gen,ht}+{\dot{S}}_{gen,fr}$$

where ${\dot{S}}_{gen,ht}\;$ denotes the entropy generation rate due to heat-transfer irreversibility and ${\dot{S}}_{gen,fr}$ represents the entropy generation rate associated with fluid friction. This decomposition provides a clear physical interpretation of the competing mechanisms responsible for thermodynamic irreversibility in internal convection systems.

### 2.2. Entropy Generation Due to Heat Transfer

At the local level, entropy generation associated with heat transfer originates from finite temperature gradients within a medium. For an isotropic material, the local volumetric entropy generation rate due to heat transfer is given by [1,2]

(2) $${{\dot{S}}_{gen,ht}}^{\prime \prime \prime }=\frac{k}{T^{2}}\;(\nabla T.\nabla T)$$

where *k* is the thermal conductivity and *T* is the local absolute temperature.Within the present conjugate formulation, Equation (2) is applicable to both the fluid and solid domains, allowing thermal irreversibility to be evaluated separately in the flowing fluid and within the pipe wall.

For system-level assessment and comparison across different operating conditions, a global heat-transfer-induced entropy generation rate is defined as

(3) $${\dot{S}}_{gen,ht}=\frac{\dot{Q}}{T_{wall,avg}}$$

where $\dot{Q}$ is the total heat transfer rate through the pipe wall and $T_{wall,avg}\;$ is the area-weighted average inner wall temperature. This global formulation provides a practical and physically consistent measure of thermal irreversibility under conjugate heat transfer conditions and is widely adopted in second-law analyses of internal flows [1,4].

### 2.3. Entropy Generation Due to Fluid Friction

Entropy generation due to fluid friction arises from irreversible momentum transport associated with viscous dissipation. At the local level, the volumetric entropy generation rate due to viscous effects is expressed as [4,14]

(4) $${{\dot{S}}_{gen,fr}}^{\prime \prime \prime }=\frac{\mu }{T}\;\Phi$$

where $\mu_{eff}$ is the effective viscosity and Φ is the viscous dissipation function, which depends on velocity gradients. For turbulent flow conditions, the effective viscosity was used in the entropy generation calculations to account for both molecular and turbulence-induced dissipation effects. Accordingly, the effective viscosity was defined as

(5) $$\mu_{eff}=\mu +\mu_{t}$$

where *μ* is the molecular viscosity and $\mu_{t}$ is the turbulent viscosity obtained from the RANS turbulence model. This approach enables turbulence-induced irreversible dissipation to be incorporated into the entropy generation analysis.

### 2.4. Bejan Number

To quantify the relative importance of heat-transfer and frictional irreversibilities, the Bejan number (Be) is introduced as a dimensionless second-law performance parameter [1,12]:

(6) $$Be=\frac{{\dot{S}}_{gen,ht}}{{\dot{S}}_{gen,ht}+{\dot{S}}_{gen,fr}}$$

Values of Be → 1 indicate that entropy generation is dominated by heat-transfer irreversibility, whereas Be → 0 corresponds to friction-dominated irreversibility. The Bejan number therefore provides a concise and physically meaningful framework for interpreting how operating and geometric parameters shift the balance between thermal and mechanical entropy generation mechanisms in conjugate heat transfer systems.

### 2.5. Implementation Within the Conjugate Framework

In the present study, entropy generation quantities are evaluated through post-processing of the converged conjugate heat transfer solutions. Temperature and velocity fields obtained from the CFD simulations are used to compute entropy generation rates according to Equations (2)–(5). Local volumetric entropy generation rates are integrated over the fluid and solid domains to obtain global entropy generation metrics, enabling consistent comparison across all investigated cases.

Importantly, no additional numerical simulations are required for the present second-law analysis. All entropy generation results are derived from the same set of validated conjugate heat transfer solutions, ensuring that observed trends are solely attributable to variations in Reynolds number, wall thickness ratio, wall thermal conductivity, and external convective conditions rather than numerical artifacts.

## 3. Materials and Methods

### 3.1. Numerical Methodology

The numerical framework employed in the present study is based on a previously validated three-dimensional conjugate heat transfer (CHT) model developed for turbulent flow in circular pipes. The governing equations for mass, momentum, and energy conservation are solved simultaneously in the fluid and solid domains, ensuring full thermal coupling between internal convection, heat conduction within the pipe wall, and convective heat exchange at the external surface.

In the present conjugate heat transfer model, the heat transfer rate was obtained from the convective boundary condition imposed at the external wall surface. The local heat flux at the outer wall was defined as

(7) $$q^{\prime \prime }=h_{out}\;(T_{w,o}-T_{\infty })\;$$

where $h_{out}$ is the external convective heat transfer coefficient, $T_{w,o}$ is the local outer wall temperature, and $T_{\infty }$ is the ambient temperature. The total heat transfer rate was calculated by integrating the local heat flux over the external wall surface:

(8) $$\dot{Q}=\int_{A_{o}}h_{out}\;\left(T_{w,o}-T_{\infty }\right)\;dA\;$$

In all cases, $T_{\infty }=300\;K$, while $h_{out}$ was varied between 5 and 100 W/m^2^K. The inlet fluid temperature was fixed at 350 K.

Turbulence effects are modeled using a Reynolds-averaged Navier–Stokes (RANS) approach, and steady-state solutions are obtained for all investigated cases.

A comprehensive description of the computational domain, boundary conditions, mesh generation, turbulence modeling, near-wall treatment, and numerical solution strategy has been reported in the authors’ previous work [13] and is therefore not repeated here for brevity. In the present study, the same numerical setup and operating conditions are retained to ensure consistency and to allow a direct second-law assessment based on the already validated conjugate heat transfer solutions.

The computational domain consisted of a straight circular pipe with conjugate heat transfer between the internal turbulent flow and the surrounding solid wall. The simulations were performed under steady-state conditions using a three-dimensional RANS-based framework. A prescribed inlet velocity corresponding to the target Reynolds number and a constant inlet temperature were imposed at the pipe inlet, while pressure outlet conditions were specified at the outlet. Convective heat exchange with the ambient environment was modeled at the outer wall surface through an external convection boundary condition. The main numerical model parameters and operating conditions are summarized in Table 1.

### 3.2. Validation and Numerical Consistency

The numerical model has been validated against established correlations and reference solutions for turbulent internal convection under conjugate boundary conditions, demonstrating good agreement in terms of total heat transfer rate and temperature distributions [13]. Numerical accuracy and solution reliability were ensured through systematic grid-independence studies, in which the computational mesh was refined until variations in key first-law quantities became negligible.

Since the present entropy generation analysis is entirely based on the converged temperature and velocity fields obtained from the validated conjugate heat transfer simulations, its accuracy directly inherits the numerical reliability of the underlying flow and heat transfer solutions. Consequently, no additional validation procedures are required specifically for the entropy generation calculations.

### 3.3. Entropy Generation Post-Processing Procedure

Entropy generation analysis is conducted as a post-processing step using the temperature and velocity fields obtained from the converged CHT simulations. Local volumetric entropy generation rates due to heat transfer and fluid friction are computed according to the governing relations presented in Section 2.

Within the conjugate framework, entropy generation due to heat transfer is evaluated separately in the fluid domain and within the solid pipe wall, enabling the contribution of each region to the overall thermal irreversibility to be quantified. Entropy generation due to viscous dissipation is computed exclusively within the fluid domain, as mechanical irreversibility is associated with momentum transport in the flowing fluid. The global entropy generation rates are obtained by integrating the local volumetric entropy generation rates over the corresponding fluid and solid domains.

### 3.4. Dimensionless Parameters and Data Reduction

To enable systematic comparison across all investigated cases, entropy generation results are analyzed as a function of Reynolds number, wall thickness ratio, wall thermal conductivity, and external convective heat transfer coefficient. The total entropy generation rate and its decomposition into heat-transfer-induced and friction-induced contributions constitute the primary second-law performance indicators.

The Reynolds number was defined using the bulk-mean inlet velocity, pipe inner diameter, and molecular fluid viscosity evaluated at the inlet temperature

(9) $$Re=\frac{\rho VD}{\mu }\;$$

where ρ is the fluid density, U is the bulk-mean inlet velocity, *D* is the pipe inner diameter, and *μ* is the molecular viscosity. In all simulations, the mass flow rate was maintained constant at the inlet boundary for each investigated Reynolds number condition.

In addition, the Bejan number is evaluated to characterize the relative dominance of thermal and mechanical irreversibilities. This dimensionless second-law parameter provides a concise measure of irreversibility partitioning and facilitates interpretation of how variations in operating and geometric parameters shift the dominant entropy generation mechanism [1,15].

## 4. Results and Discussion

In this section, the entropy generation characteristics of turbulent conjugate heat transfer in circular pipes are analyzed from a second-law perspective. The results are discussed in terms of the total entropy generation rate and its decomposition into heat-transfer-induced and friction-induced contributions, together with the Bejan number as a measure of irreversibility dominance. The effects of Reynolds number, wall thickness ratio, wall material conductivity, and external convective heat transfer coefficient are examined systematically. Unless otherwise stated, the complete numerical dataset corresponding to all 54 simulated cases is provided in Supplementary Table S1.

### 4.1. Effect of Reynolds Number on Entropy Generation

The Reynolds number plays a central role in turbulent internal flows, as it directly governs flow inertia, velocity gradients, and convective transport intensity. From a second-law perspective, variations in Reynolds number simultaneously influence thermal irreversibility associated with heat transfer and mechanical irreversibility arising from viscous dissipation. In conjugate heat transfer systems, this influence is further modulated by wall conduction and external convection, making Reynolds number a key parameter for entropy generation analysis.

To isolate the effect of Reynolds number, three representative cases were selected in which all geometric and thermal boundary conditions were kept identical, while only the Reynolds number was varied. Specifically, the wall thickness ratio, wall material conductivity, and external convective heat transfer coefficient were fixed at *δ*/*D* = 0.08, $k_{s}$ = 400 W/mK, and $h_{out}=100$ W/m^2^K, respectively, whereas the Reynolds number was varied from 5000 to 20,000. This controlled selection allows the impact of Reynolds number on entropy generation to be evaluated independently of other conjugate heat transfer parameters. The complete dataset for all simulated cases is provided in Supplementary Table S1.

**Figure 1:**
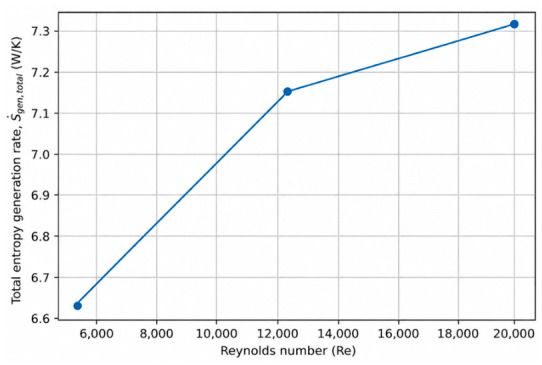
Effect of Reynolds number on the total entropy generation rate under conjugate heat transfer conditions (*δ*/*D* = 0.08, $k_{s}$ = 400 W/mK), $h_{out}$ = 100 W/m^2^K).

Figure 1 presents the variation in the total entropy generation rate with Reynolds number for the selected representative cases. As the Reynolds number increases, the total entropy generation rate exhibits a clear and monotonic increase. This trend reflects the combined effect of enhanced convective heat transfer and intensified viscous dissipation at higher flow velocities. Although increasing Reynolds number improves convective transport and reduces internal thermal resistance, it simultaneously amplifies velocity gradients near the wall, leading to higher friction-induced entropy generation. Consequently, improvements in first-law performance metrics do not necessarily translate into reduced thermodynamic irreversibility.

**Figure 2:**
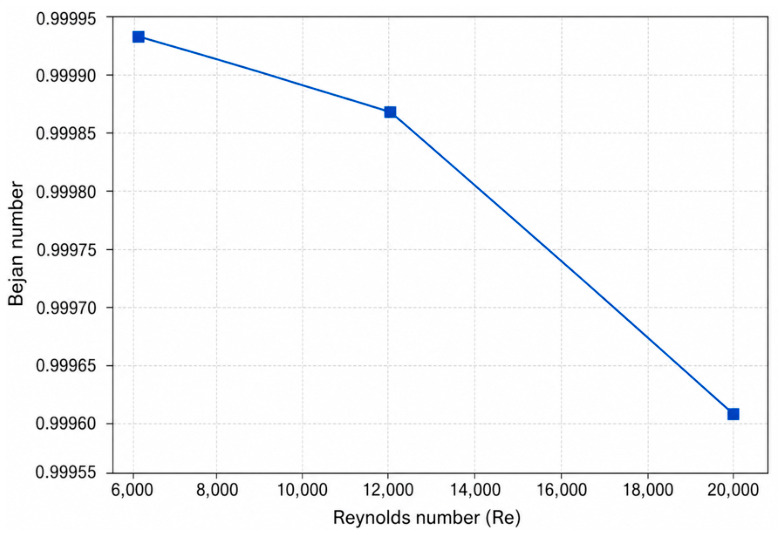
Effect of Reynolds number on the Bejan number, illustrating the relative contribution of heat-transfer and frictional irreversibilities. (*δ*/*D* = 0.08, $k_{s}$ = 400 W/mK), $h_{out}$ = 100 W/m^2^K).

To further elucidate the redistribution of irreversibility mechanisms, the variation in the Bejan number with Reynolds number is shown in Figure 2. The Bejan number decreases gradually as Reynolds number increases, indicating a growing relative contribution of frictional irreversibility to the total entropy generation. At lower Reynolds numbers, entropy generation is overwhelmingly dominated by heat-transfer irreversibility due to larger temperature differences between the fluid and the pipe wall. As Reynolds number increases, enhanced mixing and thinner thermal boundary layers reduce the relative contribution of thermal entropy generation, while frictional losses become increasingly significant.

It is noteworthy that, even at the highest Reynolds number considered, the Bejan number remains close to unity, implying that heat-transfer irreversibility continues to dominate the overall entropy generation under the present conjugate heat transfer conditions. However, the observed decreasing trend of the Bejan number clearly demonstrates that the thermodynamic cost associated with fluid friction becomes progressively more important as flow intensity increases. Similar trends have been reported for turbulent internal flows under idealized thermal boundary conditions, and the present results confirm that this fundamental trade-off persists when realistic conjugate heat transfer effects, including wall conduction and external convection, are taken into account.

It should be noted that the Bejan number remains relatively high throughout the investigated parameter range because the present conjugate heat transfer conditions are primarily governed by thermal irreversibility associated with strong temperature gradients across the coupled fluid–solid system. Although frictional entropy generation increases with Reynolds number, its contribution remains smaller than the heat-transfer-related component within the investigated operating conditions. Therefore, the Bejan number should be interpreted primarily as a qualitative indicator of irreversibility partitioning rather than as an isolated performance metric.

From a thermodynamic design perspective, the results presented in Figure 1 and Figure 2 highlight that operating at higher Reynolds numbers enhances heat transfer capability but incurs an increasing entropy generation penalty. This finding underscores the importance of balancing convective enhancement against irreversibility generation in the thermodynamic optimization of conjugate pipe systems, rather than relying solely on first-law performance indicators.

### 4.2. Effect of Wall Thickness Ratio on Entropy Generation

Wall thickness ratio (*δ*/*D*) is a key geometric parameter in conjugate heat transfer systems, as it directly controls the conductive thermal resistance of the pipe wall and governs the coupling between internal convection and external heat exchange. From a second-law perspective, variations in wall thickness alter temperature gradients within both the solid wall and the adjacent fluid region, thereby influencing the magnitude and distribution of thermodynamic irreversibilities.

To isolate the effect of wall thickness ratio, three representative cases were selected in which all operating and thermal boundary conditions were kept constant, while only *δ*/*D* was varied. In these cases, the Reynolds number was fixed at Re = 12,500, the wall material conductivity at $k_{s}\;$= 400 W/mK, and the external convective heat transfer coefficient at $h_{out}=100$ W/m^2^K. The wall thickness ratio was varied from *δ*/*D* = 0.02 to 0.08, allowing the influence of wall conduction on entropy generation to be evaluated independently of flow dynamics and external boundary effects. The complete numerical dataset for all investigated configurations is provided in Supplementary Table S1.

**Figure 3:**
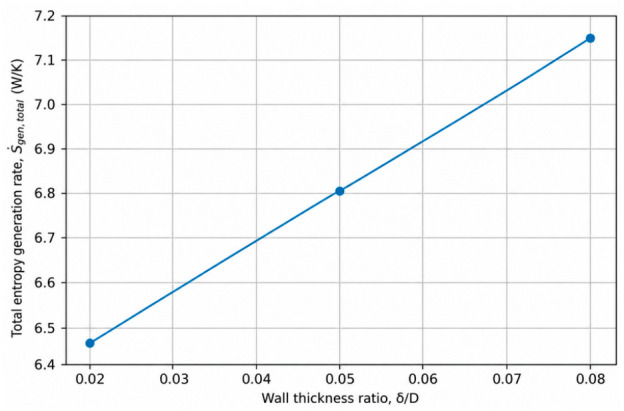
Effect of wall thickness ratio (*δ*/*D*) on the total entropy generation rate under conjugate heat transfer conditions. (*Re* = 12,500, $k_{s}$ = 400 W/mK), $h_{out}$ = 100 W/m^2^K).

Figure 3 presents the variation in the total entropy generation rate with wall thickness ratio for the selected representative cases. As *δ*/*D* increases, the total entropy generation rate exhibits a clear and monotonic increase. This behavior indicates that thicker pipe walls lead to higher overall thermodynamic irreversibility under conjugate heat transfer conditions. Since the flow field remains unchanged for all cases, the observed increase in entropy generation can be primarily attributed to enhanced conductive thermal resistance within the wall, which intensifies temperature gradients and increases heat-transfer-related entropy generation.

**Figure 4:**
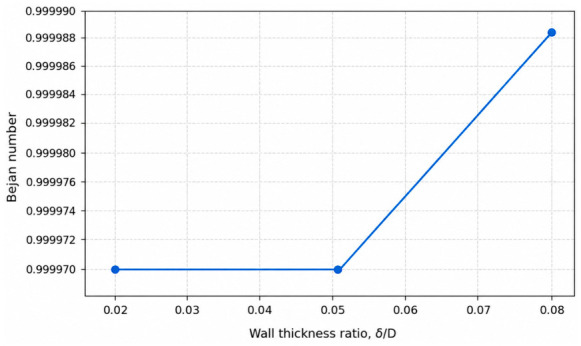
Effect of wall thickness ratio (*δ*/*D*) on the Bejan number, illustrating the redistribution of irreversibility mechanisms (*Re* = 12,500, $k_{s}$ = 400 W/mK), $h_{out}$ = 100 W/m^2^K).

The corresponding variation in the Bejan number with wall thickness ratio is shown in Figure 4. The Bejan number remains close to unity over the entire range of *δ*/*D*, indicating that entropy generation is predominantly governed by heat-transfer irreversibility. Nevertheless, a slight increase in the Bejan number is observed as wall thickness increases, suggesting that thicker walls shift the irreversibility balance marginally toward thermal mechanisms while leaving frictional contributions largely unaffected. This behavior is consistent with the fact that wall thickness does not directly influence fluid momentum transport but strongly affects thermal resistance and temperature distribution within the solid domain.

These results demonstrate that wall thickness ratio, although often treated as a secondary design parameter, plays a significant role in determining the second-law performance of conjugate pipe systems. Increasing wall thickness can impose a noticeable thermodynamic penalty by elevating heat-transfer-related entropy generation, even when flow conditions and external convection remain unchanged. Therefore, optimal design of conjugate heat transfer systems should consider wall thickness not only from structural and first-law heat transfer perspectives but also from the standpoint of entropy generation minimization.

### 4.3. Effect of Wall Material Conductivity on Entropy Generation

Wall material conductivity ($k_{s}$) plays a critical role in conjugate heat transfer systems by governing the thermal resistance of the pipe wall and controlling the coupling between internal convection and external heat exchange. From a second-law perspective, changes in wall conductivity directly affect temperature gradients within the solid domain and at the fluid–wall interface, thereby influencing heat-transfer-related entropy generation.

To examine the influence of wall material conductivity, representative cases with identical flow and geometric conditions were selected, while the wall thermal conductivity was varied. In these cases, the Reynolds number was fixed at *Re* = 12,500, the wall thickness ratio at *δ*/*D* = 0.08, and the external convective heat transfer coefficient at $h_{out}$ = 100 W/m^2^K. Two representative wall conductivities, corresponding to low- and high-conductivity materials, were considered ($k_{s}\;$= 5 and 400 W/mK). The complete dataset for all simulated configurations is provided in Supplementary Table S1.

**Figure 5:**
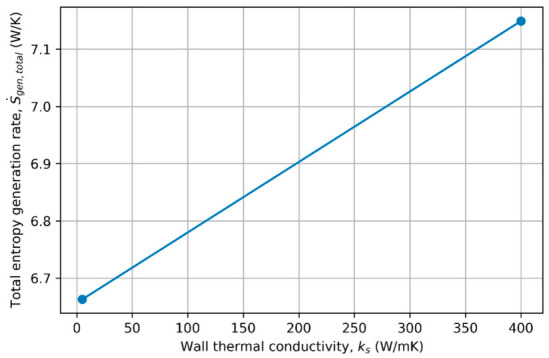
Comparison of total entropy generation rate for low- and high-conductivity pipe walls under conjugate heat transfer conditions. (*Re* = 12,500, *δ*/*D* = 0.08, $h_{out}$ = 100 W/m^2^K).

Figure 5 compares the total entropy generation rate for low- and high-conductivity walls. The results indicate that wall material conductivity has a measurable influence on the overall entropy generation under conjugate heat transfer conditions. Increasing wall conductivity enhances heat transfer through the wall and modifies temperature gradients in both the solid and fluid domains, leading to changes in heat-transfer-related irreversibility. Although higher conductivity reduces conductive resistance within the wall, the accompanying increase in heat transfer rate contributes to a higher total entropy generation in the present operating range.

**Figure 6:**
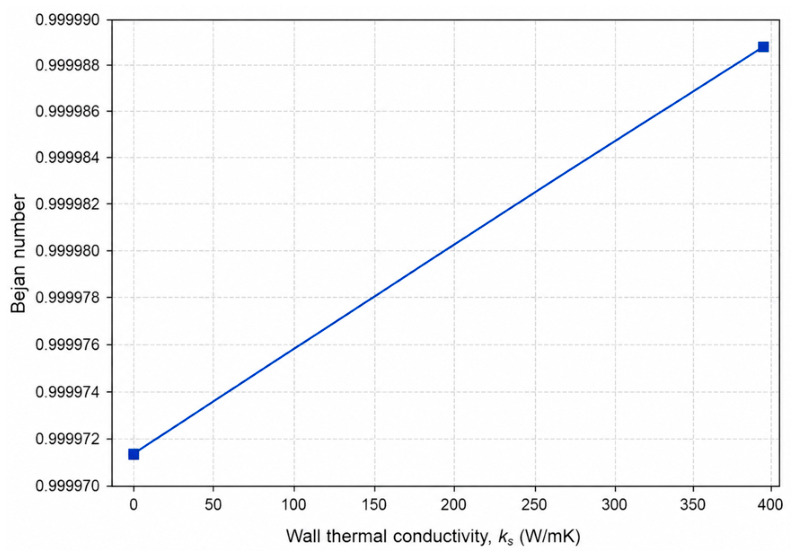
Effect of wall material conductivity on the Bejan number, illustrating the dominance of heat-transfer irreversibility. (*Re* = 12,500, *δ*/*D* = 0.08, $h_{out}$ = 100 W/m^2^K).

The corresponding variation in the Bejan number is shown in Figure 6. In both cases, the Bejan number remains very close to unity, confirming that entropy generation is dominated by heat-transfer irreversibility. The slight increase observed for the higher-conductivity wall suggests that improved thermal coupling shifts the irreversibility balance marginally toward thermal mechanisms, while frictional entropy generation remains essentially unchanged due to identical flow conditions.

Overall, these results demonstrate that wall material conductivity influences the second-law performance of conjugate pipe systems primarily through its effect on thermal transport rather than fluid dynamics. The comparison between low- and high-conductivity materials highlights the importance of material selection in controlling heat-transfer-induced irreversibility under realistic conjugate heat transfer conditions.

### 4.4. Effect of External Thermal Resistance (Biot Number) on Entropy Generation

The influence of external convection on conjugate heat transfer systems is most appropriately characterized through the external Biot number, defined as

(10) $$Bi=\frac{h_{out}.\delta }{k_{s}}$$

which represents the ratio of external convective resistance to wall conductive resistance. Unlike analyses based solely on the external heat transfer coefficient, the Biot number provides a unified measure that inherently accounts for the coupled effects of external convection intensity and wall material conductivity. From a second-law perspective, variations in the external Biot number modify the distribution of temperature gradients across the fluid–wall–environment system and therefore play a critical role in determining the magnitude and nature of entropy generation.

**Figure 7:**
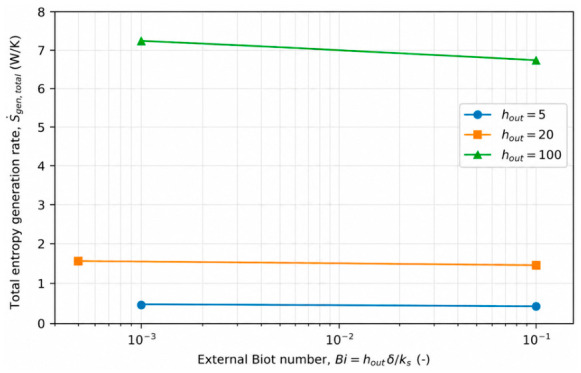
Effect of external Biot number on the total entropy generation rate under conjugate heat transfer conditions. (*Re* = 12,500, *δ*/*D* = 0.08).

To examine the effect of external thermal resistance, representative cases were selected at fixed Reynolds number (*Re* = 12,500) and wall thickness ratio (*δ*/*D* = 0.08), while the external Biot number was varied by changing the external convective heat transfer coefficient and wall thermal conductivity. Figure 7 shows the variation in the total entropy generation rate with the external Biot number. A pronounced increase in total entropy generation is observed as the Biot number increases, indicating that stronger external thermal coupling intensifies heat transfer through the wall and amplifies temperature gradients within the conjugate system. Since the flow field and associated frictional losses remain essentially unchanged for the investigated cases, the observed increase in total entropy generation is primarily attributed to enhanced heat-transfer-related irreversibility.

**Figure 8:**
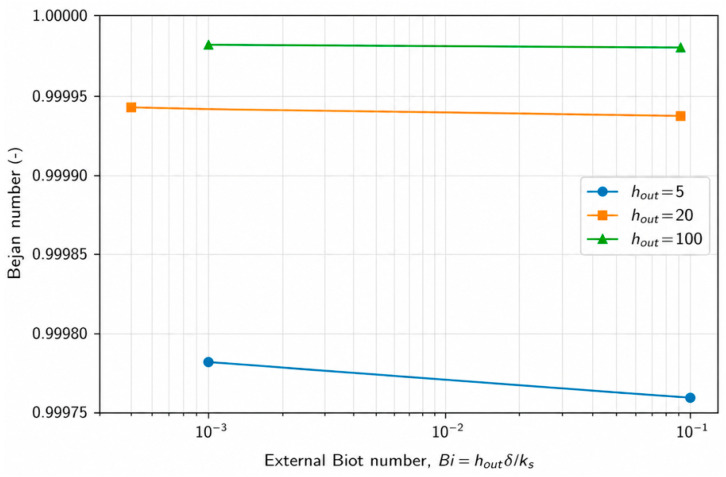
Effect of external Biot number on the Bejan number, illustrating the dominance of heat-transfer irreversibility under conjugate heat transfer conditions (*Re* = 12,500, *δ*/*D* = 0.08).

The corresponding variation in the Bejan number with the external Biot number is presented in Figure 8. Across the entire range of Biot numbers, the Bejan number remains close to unity, confirming that entropy generation is dominated by heat-transfer irreversibility under the present conjugate heat transfer conditions. Nevertheless, the gradual increase in the Bejan number with increasing Biot number reveals that strengthening the external thermal interaction shifts the irreversibility balance further toward thermal mechanisms, while the contribution of frictional entropy generation remains nearly constant. This behavior highlights the critical role of external thermal resistance in controlling the thermodynamic cost of heat transfer in ambient-coupled pipe systems.

Overall, the results demonstrate that external thermal resistance, as quantified by the Biot number, constitutes a key second-law design parameter in conjugate heat transfer applications. While increasing external convection may enhance heat removal from the system, it can also lead to a substantial increase in entropy generation and associated thermodynamic losses. Therefore, optimization of external boundary conditions should be guided not only by first-law heat transfer performance but also by entropy generation considerations to achieve thermodynamically efficient system designs.

## 5. Conclusions

In the present study, a comprehensive second-law analysis of turbulent conjugate heat transfer in circular pipes has been performed to quantify the thermodynamic irreversibilities associated with realistic fluid–solid–environment coupling. Unlike conventional first-law-based assessments, which primarily emphasize heat transfer enhancement, the present work evaluates the thermodynamic cost of heat transfer by decomposing entropy generation into heat-transfer-related and friction-related contributions and by employing the Bejan number as a diagnostic metric.

The results demonstrate that Reynolds number exerts a strong influence on total entropy generation. Increasing Reynolds number leads to a monotonic increase in total entropy generation due to intensified viscous dissipation, even though convective heat transfer is enhanced. The Bejan number decreases with increasing Reynolds number, indicating a growing relative contribution of frictional irreversibility at higher flow intensities. These findings highlight that improvements in first-law thermal performance may be accompanied by increased thermodynamic losses, underscoring the importance of balanced thermal–hydraulic optimization.

The effect of wall thickness ratio (*δ*/*D*) reveals that geometric parameters associated with wall conduction play a critical role in second-law performance. Increasing wall thickness increases total entropy generation by enhancing conductive thermal resistance and intensifying temperature gradients within the wall. Although wall thickness does not directly affect flow dynamics, it significantly alters heat-transfer-related irreversibility, demonstrating that structural design choices can impose a notable thermodynamic penalty under conjugate heat transfer conditions.

The influence of wall material conductivity ($k_{s}$) was examined through a comparative analysis of low- and high-conductivity materials. The results show that wall conductivity affects entropy generation primarily through its impact on thermal transport rather than fluid friction. Higher conductivity modifies temperature distributions and heat transfer rates, leading to measurable changes in total entropy generation and the Bejan number. These findings emphasize that material selection constitutes an effective design lever for controlling thermodynamic irreversibility in conjugate pipe systems.

The role of the external environment was assessed using the external Biot number, which provides a unified measure of external thermal resistance by accounting for both external convection intensity and wall conductivity. Increasing the external Biot number results in a pronounced increase in total entropy generation, indicating that stronger thermal coupling with the surroundings amplifies heat-transfer-related irreversibility. Across all investigated cases, the Bejan number remains close to unity, confirming the dominance of heat-transfer irreversibility; however, higher Biot numbers shift the irreversibility balance further toward thermal mechanisms. This finding highlights the importance of considering external boundary conditions as a key second-law design parameter.

Overall, the present study demonstrates that entropy generation analysis offers critical insights beyond those provided by first-law performance metrics for conjugate heat transfer systems. The results show that operating conditions, wall geometry, material properties, and external boundary conditions can all significantly influence the thermodynamic efficiency of turbulent pipe flows. From a practical standpoint, thermodynamically informed design should aim to balance heat transfer enhancement against irreversibility generation rather than pursuing maximum heat transfer alone. The findings of this study provide a framework for integrating second-law considerations into the design and optimization of realistic conjugate heat transfer applications. The presented second-law framework offers a novel thermodynamic interpretation of conjugate heat transfer systems, providing energy-relevant design insights that extend beyond conventional first-law analyses. From a practical design perspective, minimizing wall thermal resistance and carefully controlling external convection conditions are essential for reducing thermodynamic irreversibility in engineering pipe systems.

### 5.1. Limitations of the Study

The present analysis is subject to several limitations that should be acknowledged. The turbulent flow field is modeled using a steady Reynolds-Averaged Navier–Stokes (RANS) framework, and transient flow effects are not considered. Higher-fidelity turbulence modeling approaches such as Large Eddy Simulation (LES) or Direct Numerical Simulation (DNS) are beyond the scope of this study. The analysis is restricted to smooth circular pipe geometries with constant thermophysical properties. In addition, entropy generation rates are evaluated numerically through post-processing of validated conjugate heat transfer simulations without direct experimental verification. These limitations provide directions for future research toward higher-fidelity modeling and experimental validation of entropy generation in conjugate heat transfer systems.

### 5.2. Statement of Novelty

This study presents a comprehensive second-law analysis of turbulent conjugate heat transfer in circular pipe systems under realistic boundary conditions. Unlike conventional studies that rely on simplified thermal boundary assumptions or focus solely on first-law performance metrics, the present work explicitly quantifies entropy generation and irreversibility distribution within fully coupled fluid–solid–environment systems. The developed framework provides a fundamentally different thermodynamic perspective, enabling identification of dominant irreversibility mechanisms and offering deeper insight into the thermodynamic cost of heat transfer in practical engineering applications.

### 5.3. Industrial Relevance

The findings of this study provide energy-relevant insights for the design and optimization of real engineering systems such as heat exchangers, piping networks, and thermal process equipment. By identifying the dominant sources of thermodynamic irreversibility and quantifying the influence of wall properties and external convection, the results support entropy-based design strategies for improving energy efficiency and reducing thermodynamic losses in industrial thermal systems.

## Figures and Tables

**Table 1:** Summary of numerical model and operating conditions.

Parameter	Value/Description
Geometry	Straight circular pipe
Flow regime	Turbulent
Numerical model	3D steady RANS-based CHT
Reynolds number	5000–20,000
Wall thickness ratio (*δ*/*D*)	0.02–0.08
Wall conductivity (*k_s_*)	0.2–400 W/mK
External convection coefficient (*h_out_*)	5–100 W/m^2^K
Inlet temperature	350 K
Ambient temperature	300 K
Boundary condition	Convective outer wall
Fluid properties	Constant thermophysical properties

## Data Availability

The numerical data generated during the current study are available from the corresponding author upon reasonable request. No publicly archived dataset was generated.

## Supplementary Materials

The following supporting information can be downloaded at: https://www.mdpi.com/article/10.3390/e28050573/s1, Table S1: Numerical data used for entropy generation analysis.

## Abbreviations

The following abbreviations are used in this manuscript:

CHT	Conjugate Heat Transfer
RANS	Reynolds-Averaged Navier–Stokes
EGM	Entropy Generation Minimization
Re	Reynolds Number
