# Supplementary material for: Entropy Generation-Based Assessment of Thermodynamic Irreversibility in Turbulent Conjugate Heat Transfer Systems Under Realistic Boundary Conditions
Source: Entropy (Basel). 2026 May 20;28(5):573. doi: 10.3390/e28050573 (PMC13205313; doi:10.3390/e28050573)
Supplement: Supplementary file 1 [file entropy-28-00573-s001.zip › entropy-4279753-supplementary.pdf]

Table S1. Numerical data used for entropy generation analysis.

Run	Re	deltaD	delta_m	ks_WmK	hout_Wm2K	Q W	Twall,avg K	Δp_Pa	Tref_K	Sgen ht W/K	Sgen_fr_W/K
1	5000	0,02	0,001	0,2	5	117,8728	348,92	13,1051	349,854	0,338	7.38×10−6
2	5000	0,02	0,001	1	20	461,07568	347711	13,1107	349,43	1326	7.391×10−6
3	5000	0,02	0,001	5	100	2014,1905	341684	13,1107	347,509	5895	7.432×10−6
4	5000	0,02	0,001	20	5	120,91891	349521	13,1107	349,85	0,346	7.382×10−6
5	5000	0,02	0,001	200	20	470,39261	348157	13,1107	349,418	1351	7.3917×10−6
6	5000	0,02	0,001	400	100	2049,383	341,965	13,1107	347,466	5993	7.43×10−6
7	5000	0,05	0,0025	0,2	5	119,99874	347,945	13,1114	349,852	0,345	7.383×10−6
8	5000	0,05	0,0025	1	20	472,48087	346,902	13,1036	349,416	1362	7.388×10−6
9	5000	0,05	0,0025	5	100	2057,4255	340,848	13,1036	347,456	6036	7.429×10−6
10	5000	0,05	0,0025	20	5	127,77358	349,483	13,1036	349,842	0,3656	7.38×10−6
11	5000	0,05	0,0025	200	20	496,38403	348,05	13,1036	349,386	1426	7.39×10−6
12	5000	0,05	0,0025	400	100	2146,7729	341,57	13,1036	347,345	6285	7.43×10−6
13	5000	0,08	0,004	0,2	5	121,67913	346,903	13,1059	349,85	0,351	7.380×10−6
14	5000	0,08	0,004	1	20	482,3327	346,036	13,1006	349,404	1394	7.386×10−6
15	5000	0,08	0,004	5	100	2094,6236	339,988	13,1006	347,41	6161	7.429×10−6
16	5000	0,08	0,004	20	5	134,68548	349,444	13,1006	349,833	0,385	7.377×10−6
17	5000	0,08	0,004	200	20	522,54492	347,943	13,1006	349,354	1502	7.387×10−6
18	5000	0,08	0,004	400	100	2243,4353	341,179	13,1006	347,226	6576	7.433×10−6
19	12500	0,02	0,001	0,2	5	118,43955	349,155	60,7751	349,941	0,339218	8.545×10−5
20	12500	0,02	0,001	1	20	469,8749	348,622	60,7751	349,768	1,347486	8.548×10−5
21	12500	0,02	0,001	5	100	2194,0317	345,406	60,7751	348,915	6,352037	8.5698×10−5
22	12500	0,02	0,001	20	5	121,51547	349,765	60,7751	349,94	0,34742	8.545×10−5
23	12500	0,02	0,001	200	20	479,55496	349,095	60,7751	349,763	1,374	8.549×10−5
24	12500	0,02	0,001	400	100	2235,863	345,783	60,7751	348,894	6,4661	8.570×10−5
25	12500	0,05	0,0025	0,2	5	120,58628	348,18	60,736	349,94	0,346333	8.539×10−5
26	12500	0,05	0,0025	1	20	481,7306	347,82	60,736	349,762	1,385	8.544×10−5
27	12500	0,05	0,0025	5	100	2245,5485	344,582	60,736	348,889	6,5168	8.566×10−5
28	12500	0,05	0,0025	20	5	128,44124	349,741	60,736	349,936	0,367247	8.539×10−5
29	12500	0,05	0,0025	200	20	506,62048	349,039	60,736	349,749	1,451698	8.545×10−5
30	12500	0,05	0,0025	400	100	2352,5085	345,552	60,736	348,837	6,808399	8.567×10−5
31	12500	0,08	0,004	0,2	5	122,28365	347,136	60,7376	349,94	0,35226	8.538×10−5
32	12500	0,08	0,004	1	20	491,98072	346,955	60,7376	349,757	1,417996	8.544×10−5
33	12500	0,08	0,004	5	100	2290,0348	343,716	60,7376	348,867	6,662578	8.566×10−5
34	12500	0,08	0,004	20	5	135,42599	349,716	60,7376	349,933	0,387246	8.540×10−5
35	12500	0,08	0,004	200	20	533,87401	348,983	60,7376	349,736	1,529945	8.546×10−5
36	12500	0,08	0,004	400	100	2469,0528	345,322	60,7376	348,779	7,149328	8.569×10−5
37	20000	0,02	0,001	0,2	5	118,60869	349,226	136,06	349,963	0,339633	3.0636×10−4
38	20000	0,02	0,001	1	20	472,54876	348,899	136,06	349,854	1,354616	3.0646×10−4
39	20000	0,02	0,001	5	100	2253,6244	346,64	136,06	349,303	6,50134	3.0694×10−4
40	20000	0,02	0,001	20	5	121,69353	349,838	136,06	349,962	0,347857	3.06363×10−4
41	20000	0,02	0,001	200	20	482,34053	349,38	136,06	349,851	1,38063	3.0646×10−4
42	20000	0,02	0,001	400	100	2297,781	347,051	136,06	349,29	6,620874	3.06952×10−4
43	20000	0,05	0,0025	0,2	5	120,76174	348,25	135,976	349,963	0,346767	3.06173×10−4

44	20000	0,05	0,0025	1	20	484,54353	348,099	135,976	349,85	1,39197	3.06272×10−4
45	20000	0,05	0,0025	5	100	2308,0569	345,823	135,976	349,286	6,674099	3.06766×10−4
46	20000	0,05	0,0025	20	5	128,64028	349,818	135,976	349,96	0,367735	3.06175×10−4
47	20000	0,05	0,0025	200	20	509,73379	349,34	135,976	349,842	1,459134	3.06279×10−4
48	20000	0,05	0,0025	400	100	2421,2354	346,882	135,976	349,251	6,98	3.06797×10−4
49	20000	0,08	0,004	0,2	5	122,4641	347,205	135,987	349,962	0,352714	3.06198×10−4
50	20000	0,08	0,004	1	20	494,91516	347,235	135,987	349,847	1,425303	3.06299×10−4
51	20000	0,08	0,004	5	100	2355,0832	344,957	135,987	349,272	6,827179	3.06803×10−4
52	20000	0,08	0,004	20	5	135,64745	349,797	135,987	349,958	0,387789	3.06202×10−4
53	20000	0,08	0,004	200	20	537,33332	349,3	135,987	349,834	1,538315	3.06310×10−4
54	20000	0,08	0,004	400	100	2544,846	346,713	135,987	349,213	7,339921	3.06855×10−4

Sgen_total_W/K	Bejan
0,337829	0,999978
1,326039	0,99999443
5,8949	0,99999874
0,345964	0,999979
1,35109388	0,999995
5,99296	0,99999876
0,344887	0,999979
1,362017	0,9999946
6,036276	0,99999877
0,3656	0,99998
1,426	0,9999948
6,285	0,9999988
0,350766	0,999979
1,393888	0,9999947
6,160883	0,99999879
0,385435	0,9999809
1,501819	0,9999951
6,575545	0,99999887
0,339303	0,999748
1,347572	0,999937
6,352123	0,9999865
0,347506	0,999754
1,37409	0,999938
6,466173	0,9999867
0,346419	0,999754
1,385085	0,999938
6,516887	0,9999869
0,367332	0,999768
1,451784	0,999941
6,808485	0,9999874
0,35235	0,999758
1,418081	0,99994
6,662664	0,999987
0,387331	0,99978
1,530031	0,999944
7,149414	0,999988
0,339939	0,999099
1,354922	0,999774
6,50165	0,999953
0,348163	0,99912
1,38094	0,999778
6,621181	0,9999536
0,347074	0,999118

1,392277	0,99978
6,674406	0,999954
0,368041	0,999168
1,45944	0,99979
6,980304	0,999956
0,35302	0,999133
1,42561	0,999785
6,827486	0,999955
0,388095	0,999211
1,538621	0,999801
7,340228	0,999958
